# Real-Time Polypyrrole Electrodeposition Kinetics Monitored by Surface Plasmon Resonance: Impact of the Deposition Method and Dopamine Copolymerization

**DOI:** 10.1021/acs.langmuir.5c01765

**Published:** 2025-07-18

**Authors:** Kieke de Boer, Karin Schroën

**Affiliations:** Laboratory of Food Process Engineering, 4508 Wageningen University & Research, Bornse Weilanden 9, 6708 WG Wageningen, Netherlands

## Abstract

The modification of surfaces with conductive polymers, such as polypyrrole, creates functional materials that can be applied in a wide range of fields. To optimally utilize polypyrrole materials, in particular those produced through electrodeposition, we require knowledge of film properties and how these are affected by the deposition technique. In work done by others, the focus is mostly on the layer properties that are achieved and not on the early stages of synthesis that ultimately determine these properties. Here, we evaluate the role of electrodeposition techniques and dopamine on the early stages of polymerization kinetics of polypyrrole thin films (<30 nm) through real-time analysis with surface plasmon resonance (SPR). Electrochemical polymerization was performed through galvanostatic, potentiostatic, pulsed galvanostatic, and pulsed potentiostatic deposition, varying the applied current or potential, in the absence and presence of dopamine. The results reveal that the polymerization speed is technique-dependent and connected to the measured potential or current. Polymerization is limited by pyrrole radical formation, which can be partially mitigated by reaching the threshold potential (±0.4 V). The polymerization speed increases over the synthesis time due to the decreasing oxidation potential of larger pyrrole structures. Dopamine copolymerization catalyzes the initial pyrrole radicalization but shifts the polymerization location from surface- to solution-based, therewith reducing the polymerization speed on the surface. The investigated deposition conditions resulted in pronounced differences in composition, structure, thickness, and visual appearance of the films. The real-time evaluation carried out in this paper provides insights into the effect of the deposition technique and dopamine on the initial polymerization reactions, polymerization speed, and controllability. Connecting these insights in polypyrrole polymerization with film properties is essential for the utilization of polypyrrole as a smart material in various fields, e.g., sensors, batteries, or biomaterials.

## Graphical abstract

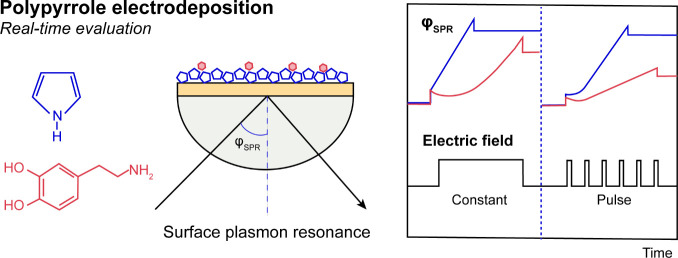

## Introduction

Electrically conductive polymers, such as polypyrrole, are relevant for the development of supercapacitors and batteries, (bio)­sensors, anticorrosion coatings, and biomaterials for drug delivery or artificial muscles, among others.^1–6^ Polypyrrole stands out for its high conductivity, thermal and chemical stability, biocompatibility, low costs, and relatively easy polymerization options. Polymerization can be performed through chemical and electrochemical routes, with the latter one being a versatile and facile method for controlled film formation, directly on the surface of the electrode material.^7–9^

The polymerization of polypyrrole, according to Diaz’s mechanism, is initiated by oxidation, whereby a primary radical cation is formed (Figure 1).^10,11^ The coupling between two radicals results in the formation of a positively charged dimer, which stabilizes through the loss of two protons. The neutral dimer can be oxidized to a radical cation again, thereby allowing coupling to other radical cations, leading to chain propagation. The continuous formation of radical cations through oxidation introduces charge carriers along the polypyrrole backbone. To compensate for these charges, anions move from the solution to the polymer structure, which is known as doping.^12^ For a detailed overview of the mechanism, we refer to the work of others.^10,11^

**Figure 1:**
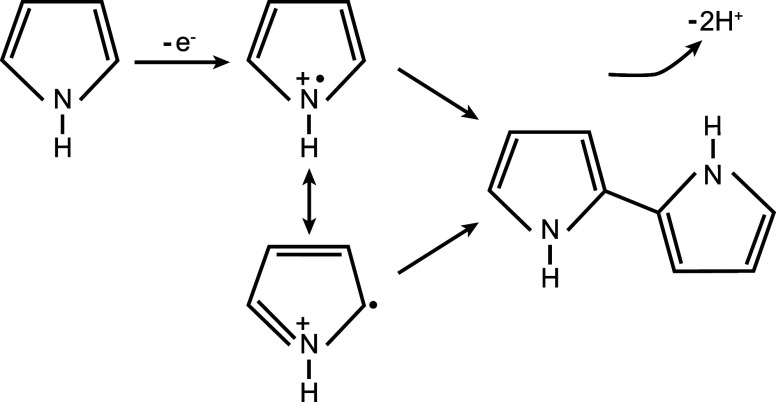
Initial reactions during polypyrrole electropolymerization: pyrrole oxidation and formation of the dimer.

Since the first synthesis of polypyrrole reported in the 1970s, many articles have reported different routes and conditions for electrodeposition, which influence structural and functional properties.^8,10,13^ For tailor-made functional polypyrroles, we require knowledge of the effect of synthesis conditions on polymerization and ultimately on film properties. Films can be tuned by adapting a wide variety of factors, such as the pyrrole concentration,^14–17^ type and concentration of dopant,^7,10,14,16–21^ solvent type,^22^ electrode material,^10,23^ temperature,^16,17,24^ and pH.^25–27^ Moreover, the electrodeposition technique and its strength and duration strongly influence the polypyrrole film properties.^7,9,16,17,23,27–32^

Generally, three modes of electrodeposition are applied: galvanostatic, potentiostatic, and cyclic voltammetry. Galvanostatic polymerization uses constant current density; potentiostatic polymerization uses constant potential; and cyclic voltammetry is a dynamic technique, where the potential changes in a cyclic manner. Over the last years, pulsed potentiostatic and pulsed galvanostatic polymerization have received more attention due to their positive effects on electrical conductivity, stability, and reduced chain defects.^32–34^ For these methods, the current or potential is fixed during an on-time period, followed by an off-time period during which the current flow is minimized, which has been reported to create more space between polymers and allow better ionic transfer within materials.^34^ In general, polymerization is evaluated based on the final polymer properties instead of synthesis kinetics. In the current paper, we use galvanostatic, potentiostatic, pulsed galvanostatic, and pulsed potentiostatic polymerization and evaluate polypyrrole growth in the early stages of synthesis to analyze the basis from which the properties of polypyrrole films stem.

Currently, adhesion of polypyrrole coatings onto the electrode surface is still poor due to the lack of strong molecular interactions with the electrode, which stands in the way of practical applications. Adhesion can be improved by the incorporation of polydopamine, which mimics bioadhesive moieties of marine mussels. Strong interactions between (poly)­dopamine and the electrode surface and between (poly)­dopamine and the polypyrrole chains are formed through hydrogen-bonding and π-π interactions with catechol and amine groups.^35–37^ Polydopamine can be formed from dopamine monomers through self-polymerization at slightly alkaline pH (8.5) or under oxidizing conditions^38,39^ on a variety of materials.^40^ Co-electropolymerization of dopamine with pyrrole into a polydopamine–polypyrrole coating has been shown to lead to enhanced stability and diminished polypyrrole detachment.^37,41,42^ Additionally, it is reported that dopamine has a catalytic effect on polypyrrole deposition. Dopamine first oxidizes to dopaquinone or dopaminochrome, which reduces the conjugation energy of pyrrole rings upon interaction and facilitates pyrrole radicalization.^39,41,43^ The incorporation of dopamine simultaneously creates a more ordered polymer structure, allowing more efficient charge transport and reducing the risks of side reactions.^42,43^

Here, we focus on the early stages of polypyrrole deposition and report on the role of dopamine and the deposition technique in the initial film formation kinetics. Synthesis is performed in a surface plasmon resonance (SPR) system, allowing real-time evaluation of the growth on a gold surface,^44–46^ for which we report the polymerization speed and controllability as well as the polypyrrole film properties obtained. The improved understanding of polypyrrole growth behavior that was achieved leads to routes for controllable polypyrrole formation for a wide range of potential applications.

## Materials and Methods

### Chemicals

The pyrrole monomer was purchased from Sigma-Aldrich, distilled, and stored in the dark at −20 °C until usage. Sodium dodecylbenzenesulfonate (NaDBS), which was used as a dopant, and dopamine hydrochloride (≥98%), which was used as an adhesive, were both purchased from Sigma-Aldrich. All dilutions were made with ultrapure Milli-Q water obtained from a Millipore Milli-Q system (18.2 MΩ).

### Electrochemical Polymerization

Synthesis of polypyrrole was performed through electrodeposition in an Autolab ESPRIT SPR instrument (Kinetic Evaluation Instruments, Netherlands) in combination with an electrochemical cell. A polarized laser light (670 nm) was directed to the bottom of the sensor through a glass hemicylinder prism, and the reflected light was monitored with a diode detector. The electrochemical cell used a three-electrode setup with a SPR sensor, a gold-coated glass disk (SSENS, Netherlands) as the working electrode (±0.06 cm^2^), a platinum rod as the counter electrode, and a removable Ag/AgCl wire as the reference electrode. All reported potentials are measured against the AgCl reference electrode calibrated at −0.1 V vs Ag/AgCl (3 M KCl). An overview of the system can be found in Figure S1.

The gold working electrode was first stabilized in 10 mM phosphate buffer (pH 6) for 20 min, with 100 μL of fresh solution injected every 120 s. The synthesis solution was prepared with 0.1 M pyrrole, 0.1 M NaDBS, and 0.02 M dopamine hydrochloride in a 10 mM phosphate buffer (pH 6). After 100 μL of synthesis solution was injected onto the gold surface, the shift in signal was monitored for 180 s. Polymerization was initiated by starting electrodeposition with a connected potentiostat (Ivium, Netherlands). Electrodeposition was performed under potentiostatic (0.4, 0.5, and 0.6 V) and galvanostatic (40, 50, and 60 μA) conditions until a 1.5 mC charge passed through the system. Dynamic polymerization was performed through pulsed potentiostatic and galvanostatic techniques. For pulsed potentiostatic deposition, the potential was switched between the on mode (0.4, 0.5, and 0.6 V, 0.2 s) and off mode (0 V, 2 s) for 100 cycles. The pulsed galvanostatic technique varies the current between the on mode (50, 100, and 150 μA, 0.2 s) and off mode (0 μA, 2 s) for 150 cycles. After polymerization, water was flushed through the system for 10 min, after which 10 mM phosphate buffer was injected to assess the final polymer growth.

The polymerization potentials were selected close to the oxidation potential of pyrrole^47^ and kept below the overoxidation potential.^7,12^ The currents for galvanostatic deposition were chosen to achieve similar potentials, while higher currents were required for pulsed galvanostatic deposition to observe any polymerization. The on mode for pulsed techniques was kept at 10% of the off mode in line with suggestions by others^32,33^ while operating at the lower time limit of the potentiostat.

The effect of dopamine was evaluated either using a prelayer or as part of the synthesis liquid for copolymerization. The prelayer was made by injecting 1 g/L dopamine in 100 mM Tris hydrochloride (pH 8.5) for 10 min. Next, the surface was rinsed, and the polymerization protocol described above was followed. For effects of copolymerization, the molar dopamine/pyrrole ratio in the synthesis solution was varied between 0, 0.1, 0.2, and 0.3, a range within the optimal ratio reported by others.^41,43^

### Data Analysis

SPR angles were continuously monitored (1 measurement/second). The data sets were first normalized by setting the measured baseline as zero. The effect of pyrrole was determined from the angle shift obtained upon injection, and the final polymer growth was derived from the difference between the final and initial baselines in phosphate buffer. The polymerization speed, described as SPR angle shift (mdeg/m°)/second, was calculated with a Δ*t* of 1 s for all galvanostatic and potentiostatic depositions, 20 s for pulsed depositions in the presence of dopamine, and 5 s for pulsed depositions without dopamine, to avoid inclusion of the decreasing SPR angle during the off period. All experiments were repeated at least twice, from which average kinetic curves and polymerization speeds were determined.

The potentiostat data were utilized to plot the potential/current over time, which served as a basis to analyze polymerization speed with respect to the potential/current. The time at which the speed was determined was the time closest to the measured potential/current. For the pulsed depositions, the potential/current peaks (on period) were extracted first and, consequently, plotted against the matching polymerization speed. To remove the impact of the refractivity index change, a consequence of the electric field, the first 1 or 2 data points were excluded for pulsed galvanostatic and galvanostatic deposition, respectively.

### Structural Characterization

Structural characteristics of the polypyrrole films were assessed by scanning electron microscopy (SEM). Images were taken at 10000× and 25000× magnification with a Magellan 400 microscope (FEI Company, Hillsboro, OR, U.S.A.), operating at 2.00 kV beam energy and 13 pA beam current. The thickness was determined using an EP4 imaging ellipsometer (Accurion, Germany) in a wavelength range between 630 and 730 nm at a fixed angle of incidence of 50° in air at room temperature. The acquired Δ and Ψ values were fitted with the EP4 software (Nanofilm) using a multilayer model. The model consisted of the gold-coated SPR sensor and the polypyrrole polymer layer, which was described using a Cauchy model (*A* = 1.450 and *B* = 4500). Each sample was measured at two locations in the middle of the polypyrrole film.

### Surface Chemistry

The chemical composition was determined using X-ray photoelectron spectroscopy (XPS, JPS-9200, Joel, Ltd., Japan). All samples were analyzed with a focused monochromated Al K X-ray source with a spot size of 300 μm under ultra-high-temperature (UHT) conditions (base pressure of 3 × 10^–7^ Pa). Radiation was set at 12 kV and 20 mA with an analyzer energy pass of 10 eV. In addition to wide scans, C 1s and N 1s narrow scans were measured, which were corrected using a Shirley background in CasaXPS.

## Results and Discussion

### Electrochemical Deposition in the SPR System

For those unfamiliar with SPR, we first explain some basic aspects of this technique. Polypyrrole polymerization can be followed by a shift in the resonance angle (SPR angle), which reacts to changes in the refractive index of the gold sensor electrode (from now on called the gold electrode) and is therefore sensitive to polymer formation. A schematic overview of a typical curve obtained during polymerization is shown in Figure 2. First, a stable baseline is created with 10 mM phosphate buffer (1). Upon injection of pyrrole solution (2), the SPR angle increases with 1005 ± 65 m°, mainly because of a change in refractive index. When electrochemical polymerization (3) is started, the SPR angle increases over time. Often, a sudden increase can be seen at the start of synthesis, which is likely related to a change in the gold electrode properties and refractive index close to the surface due to the presence of the electric field.^48,49^ Polymerization was stopped by discontinuing the electric field (4), whereby the total synthesis duration was limited by the maximum angle shift. Consequently, the SPR angle slightly decreases due to changes in the refractive index and the properties of the gold electrode. In the work of Bailey et al.,^44^ the SPR angle kept increasing after stopping synthesis due to the changing optical properties of the polypyrrole film, which we did not observe in our work. We can safely assume that we are predominantly measuring the adhered polypyrrole layer and not the smaller dissolved products since the distance between the start and end of polymerization (points 2 and 4) is always similar to the distance between the initial and final baseline in buffer (points 1 and 5) (Figure S2). The shift in SPR angle during synthesis can be related to the extent of polymerization and used as a measure of the polymerization speed.^50,51^

**Figure 2:**
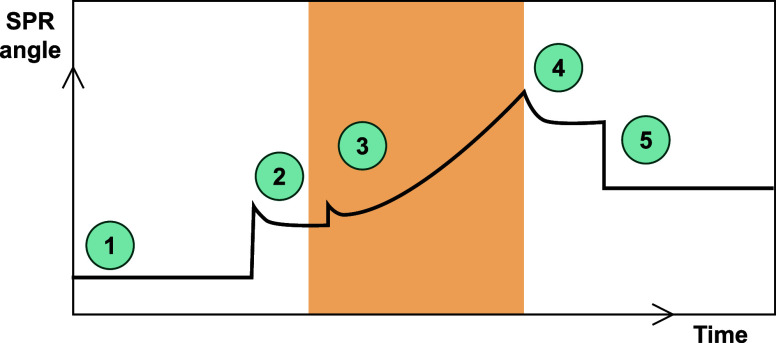
Schematic SPR angle shift during electrodeposition of polypyrrole on the gold surface: (1) baseline in buffer, (2) injection of pyrrole, (3) electrodeposition, (4) discontinuing electric field, and (5) baseline in buffer. Please note that in graphs 3–5, only the orange section of the SPR curve is shown.

### Polypyrrole Growth: Influence of the Deposition Technique

The SPR kinetic curves and the corresponding measured current or potential over time are shown in Figure 3A1–D1 and A3–D3, for the four electrodeposition techniques. Based on these results, the polymerization speed is calculated and plotted against time (Figure 3A2–D2) and against the measured current or potential (Figure 3A4–D4). All results represent the orange section of Figure 2. Overall, the techniques show pronounced differences in SPR angle development and hence layer development, final polypyrrole growth, and polymerization speed. Pulsed techniques show slower progress due to the off period, resulting in a lower overall polymerization speed. Faster polymerization can be seen for the static techniques, with the highest speed found for potentiostatic growth at 0.6 V. For all techniques, at the start, the polymerization speed is relatively low compared to the maximum speed reached. This typically points toward limitations during initiation, mainly polypyrrole radical formation.^10,11^ Faster polymerization occurs once the dimer is formed as the unpaired electron is now delocalized over two rings, making oxidation easier. Over the duration of synthesis, the decreasing oxidation potential of larger pyrrole structures further increases the polymerization speed.^13^ For all techniques, the polymerization speed correlates to the measured current or potential, albeit differently, showing that the technique influences polymerization progress as well. The four methods are now discussed one by one to highlight specific aspects.

**Figure 3:**
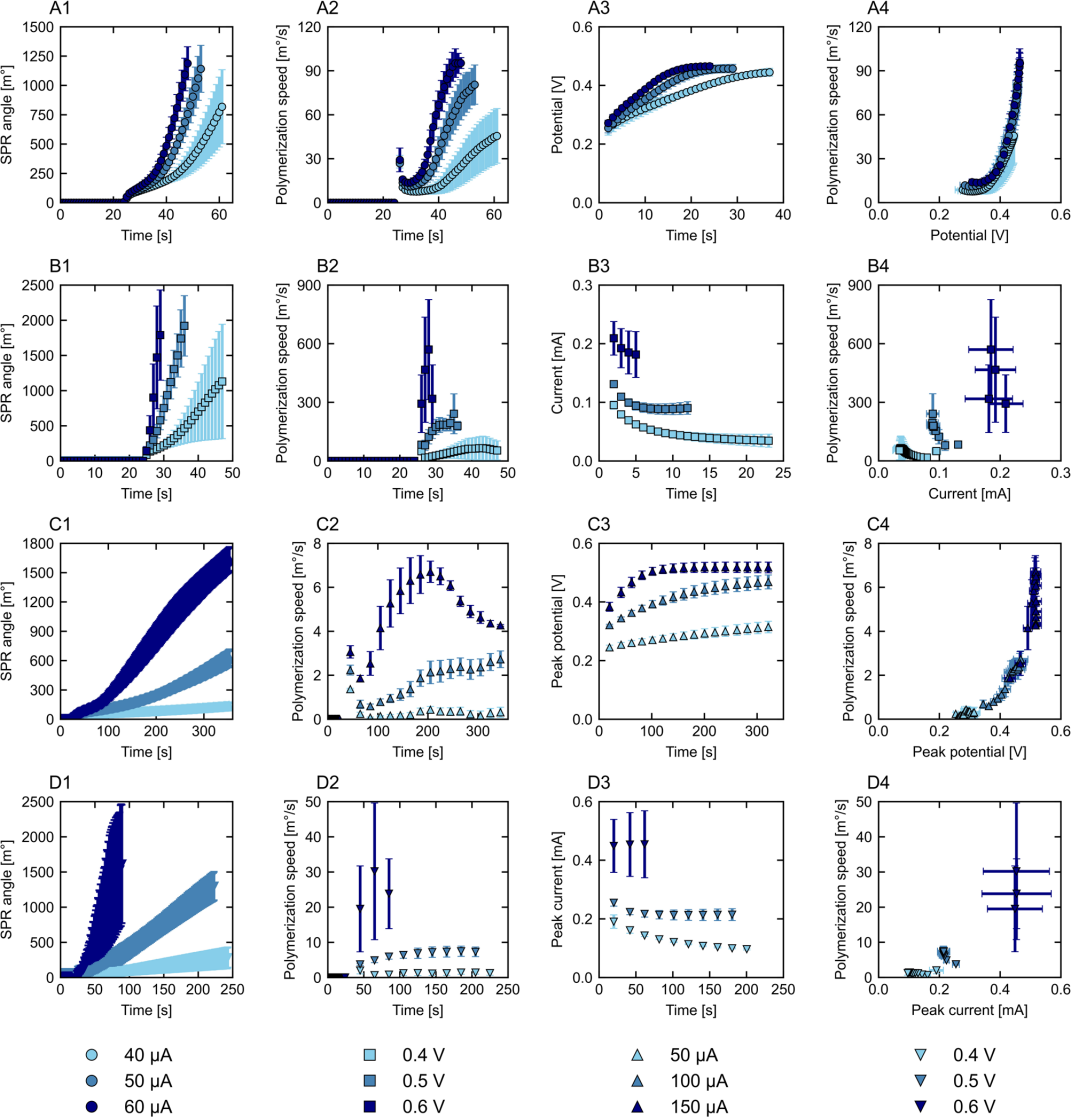
SPR curves (1), polymerization speed (2), potential/current over time (3), and polymerization speed over measured potential/current (4) for galvanostatic (A, ⬤), potentiostatic (B, ■), pulsed galvanostatic (C, ▲), and pulsed potentiostatic (D, ▼) deposition.

For galvanostatic growth (Figure 3A), a constant current is applied, which should result in a constant polymer formation rate, when the electrical conductivity remains unchanged.^10^ Applying the current causes a small jump in the SPR angle, especially visible in the polymerization speed (Figure 3A2), caused by the refractive index changing in response to the electric field. Afterward, the actual polymerization starts at a low polymerization speed, which rapidly increases and stabilizes at the end of the synthesis period, which is in line with the development of the measured potential (Figure 3A3). Overall, the polymerization proceeds faster at higher applied currents due to the increased rate of pyrrole monomer oxidation. In Figure 3A4, the measured potential is plotted against the polymerization speed, showing that the initial polymerization speed is potential-dependent. Above ±0.39 V, a positive linear correlation is found between the polymerization speed and the measured potential, suggesting a transition between limited and free pyrrole radicalization. This threshold value is consistent with previously reported required potentials for polypyrrole growth in the presence of dopamine.^41,52^ For synthesis at 40 μA, the measured potential remains below 0.39 V relatively long, resulting in a lower polymerization speed and final growth, although the passed charge was constant throughout all galvanostatic depositions (1.5 mC). When the deposition is compared at 50 and 60 μA, the angle shift and measured potential are quite similar, although the faster increase in potential for the 60 μA deposition results in the more rapid polymerization on the surface from the synthesis start. As the measured potential at all currents stabilizes around the same value while the polymerization speed remains different, we expect that even small potential differences affect the polymerization. For longer synthesis times, we expect a constant polymerization speed and a first-order relation between the applied current and speed.^10,17,53^ Additionally, the measured potential is expected to decrease again due to the presence of more oligomers compared to monomers, which have a lower oxidation potential.^28^ These results highlight the importance of reaching the oxidation potential to accelerate initial polymerization reactions. Overall, galvanostatic electrodeposition proved to be greatly controllable and predictable during the measured polymerization initiation phase.

For potentiostatic deposition (Figure 3B), a fixed potential is applied and the corresponding current is followed over time (Figure 3B3). Polymerization is faster (±10 s) compared to galvanostatic growth, especially at 0.5 and 0.6 V, and is potential-dependent. Currents around 100 and 200 μA are measured (Figure 3B3), thus largely exceeding the set currents for galvanostatic deposition. The high initial current value has been associated with several processes, such as the formation of a double layer, oxidation of the electrode surface, and monomer oxidation, after which the current decreases and stabilizes.^14,28,54,55^ At 0.5 V, the polymerization speed becomes stable after a few seconds, indicative of constant radicalization and chain propagation, which has not yet been reached for the galvanostatic depositions. However, relatively high standard deviations in the SPR angle shift are noticeable for 0.4 and 0.6 V (Figure 3B1), indicative of less controllable polymerization compared to the galvanostatic method. From the galvanostatic deposition, we observed an increasing polymerization speed above 0.39 V. We expect that, at 0.4 V, the reaction remains at the border of free and limited pyrrole radicalization, causing the experimental deviation. Variation at 0.6 V is likely the result of fast and less controlled pyrrole radicalization due to the high current density combined with the limited measurement window of the SPR system. Stable polypyrrole polymerization has been performed at higher potentials^14,52^ for longer synthesis times but in a different setup. We find similar unstable results for pulsed potentiostatic deposition at 0.6 V, emphasizing that the applied potential determines the controllability of the polymerization and therefore suitability for this system.

Both pulsed techniques make use of an on mode, during which polymerization occurs, and an off mode, when polymerization is minimized. The switch between these modes is clearly visible by alternating potential and current peaks, leading to a fluctuating result in the SPR angle shift (Figure S3). For the calculation of the polymerization speed, we use a larger time step (Δ*t* = 20 s) compared to the static depositions (Δ*t* = 1 s) to avoid negative values and represent the overall growth. In Figure 3, we only show the peak potential and peak current during these depositions. Polypyrrole polymerization takes longer for both pulsed techniques due to the off period, which consequently reduces the polymerization speed by an order of magnitude. For both pulsed depositions, the polymerization speed is determined by the applied current or potential, similar to the static depositions. Pulsed galvanostatic deposition (Figure 3C) shows an increasing polymerization speed over time, which connects well with the measured peak potential. For 100 and 150 μA, the linear increase in polymerization speed starts at peak potentials of 0.37 and 0.38 V, respectively, closely matching the value for galvanostatic deposition. For pulsed deposition at 50 μA, the growth is close to none, unlike regular galvanostatic deposition at this current. We hypothesize that the pulsed method does not allow sufficient time to reach the required potential to overcome limitations of pyrrole radicalization, as approximately 10 s was required in the static deposition and solely 0.2 s pulses were applied. At the end of the 150 μA experiment, the polymerization speed declines, while the peak potential remains constant, which is only seen for this condition. As the peak potential remains constant, we hypothesize that the polymerization speed declines due to mass transport limitations, resulting in insufficient monomers and dopants at the surface. Alternatively, a partial switch from surface- to solution-based polymerization could have occurred. For pulsed potentiostatic deposition (Figure 3D), the peaks of the on period decrease over synthesis time, eventually stabilizing at different currents for the three potentials, matching the results seen for potentiostatic deposition (Figure 3D3). The polymerization speed can be controlled at 0.4 and 0.5 V, as shown in Figure 3D1 and D2, unlike the 0.6 V experiment for which only a part of the cycles could be performed to stay in the measurement window of the SPR, resulting in a high standard deviation as discussed previously. Polymerization with 0.5 V pulses seems to be a suitable method for polypyrrole growth, taking into account the required oxidation potential for reduced pyrrole oxidation limitations and controllability of the growth. In general, the pulsed depositions might be easier to control compared to the static methods due to the reduced polymerization speed.

Overall, we can conclude that the electrodeposition technique influences the growth of polypyrrole, including its controllability. Pulsed electrodepositions show slower polymerization speeds compared to the static methods at the same current or potential due to the presence of the off period. Good controllability over polypyrrole polymerization is generally achievable for all deposition methods as long as the potential remains below 0.6 V. The lower potential limit is determined slightly below 0.4 V, which is sufficient to overcome limitations during pyrrole radical formation.

### Polypyrrole Growth: Influence of Dopamine

The role of dopamine in the initial polymerization steps was further explored by copolymerization of dopamine and pyrrole at varying molar ratios and through the addition of a (poly)­dopamine prelayer. Copolymerization ( 0.02 mM dopamine) was studied for all deposition techniques, of which the results can be found in Figure 4. Additional information on the potential/current over time and the polymerization speed over measured potential/current can be found in Figures S4 and S5 of the Supporting Information. The polymerization speed is lower for all deposition techniques when dopamine is used, although the effect was limited for potentiostatic deposition. Furthermore, the current increased when dopamine was used for (pulsed) potentiostatic depositions, while the measured potential for (pulsed) galvanostatic depositions is lower. This indicates that the resistance of the system decreases with the addition of dopamine, which should facilitate the oxidation processes during polymerization, as was previously reported for deposition through potentiostatic,^37,41,52^ galvanostatic,^42^ and cyclic voltammetry^52,56^ techniques. That would also be expected, given the catalytic role of dopamine in polypyrrole polymerization through the formation of dopamine radicals and as an electron mediator.^37,41,42,52^ In the work of Behan et al.,^52^ the addition of dopamine increased the polypyrrole layer thickness and growth rate during potentiostatic deposition when measured with QCM, unlike our SPR data that shows reduced polypyrrole polymerization in the presence of dopamine. Even though the used dopamine to pyrrole ratio was the same in the work of Behan et al., differences in pH and potential are known to influence polydopamine–polypyrrole films, which could explain the differences.^27^ We expect that, in our case, dopamine does have a catalytic role but that formation of polypyrrole–polydopamine structures happens in the liquid close to the surface rather than on the gold electrode.^42^ The extent of polymerization in the solution and therefore the impact on the measured surface polymerization depends on the chosen deposition technique.

**Figure 4:**
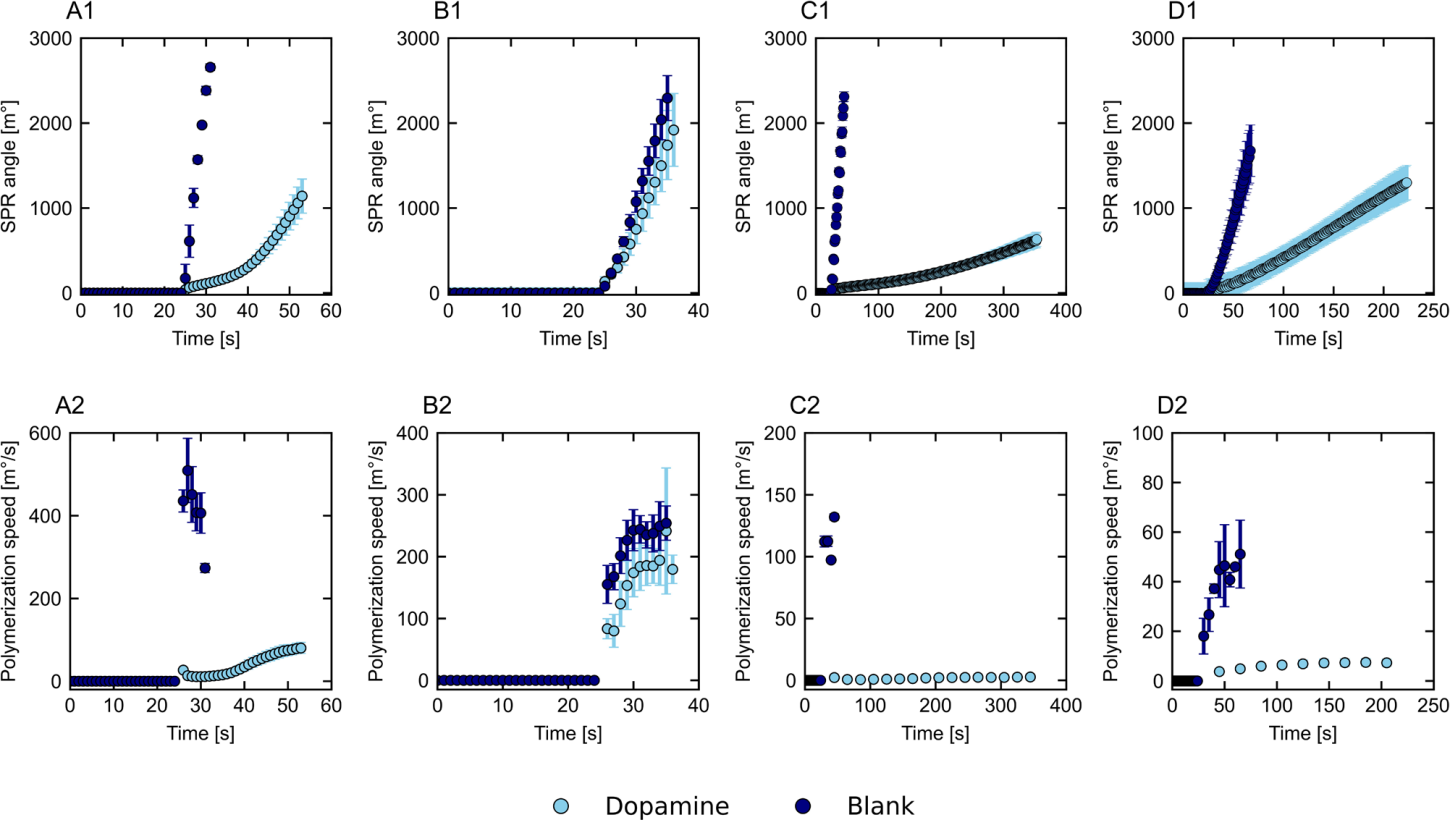
Effect of dopamine on the SPR curves (1) and polymerization speed over time (2) for galvanostatic (50 μA, A), potentiostatic (0.5 V, B), pulsed galvanostatic (100 μA, C), and pulsed potentiostatic (0.5 V, D) deposition.

For galvanostatic deposition at 50 μA, the effect of the dopamine/pyrrole ratio was studied, which can be seen in Figure 5A. The ratio influences the polymerization speed, stability, and development of the potential over time. For the 0.1 ratio, the polymerization speed proceeded slowest, and at a ratio of 0.2 or 0.3, the speed was similar, albeit that the 0.2 ratio showed less variation. The limited difference between these ratios matches earlier observations by Kim et al.,^41^ where the current and mass over time during polymerization were similar. Potential–time curves reveal that the measured potential is lower when dopamine is used, with the lowest values for the 0.2 and 0.3 ratios. This indicates reduced system resistance, connecting to the catalytic effect of dopamine.^37,41^ Notably, the measured potential for the depositions with dopamine approaches that of the blank, indicating that the polymerization might become similar over longer synthesis times. This supports the hypothesis that the catalytic role of dopamine is most relevant during the initial polymerization steps.

**Figure 5:**
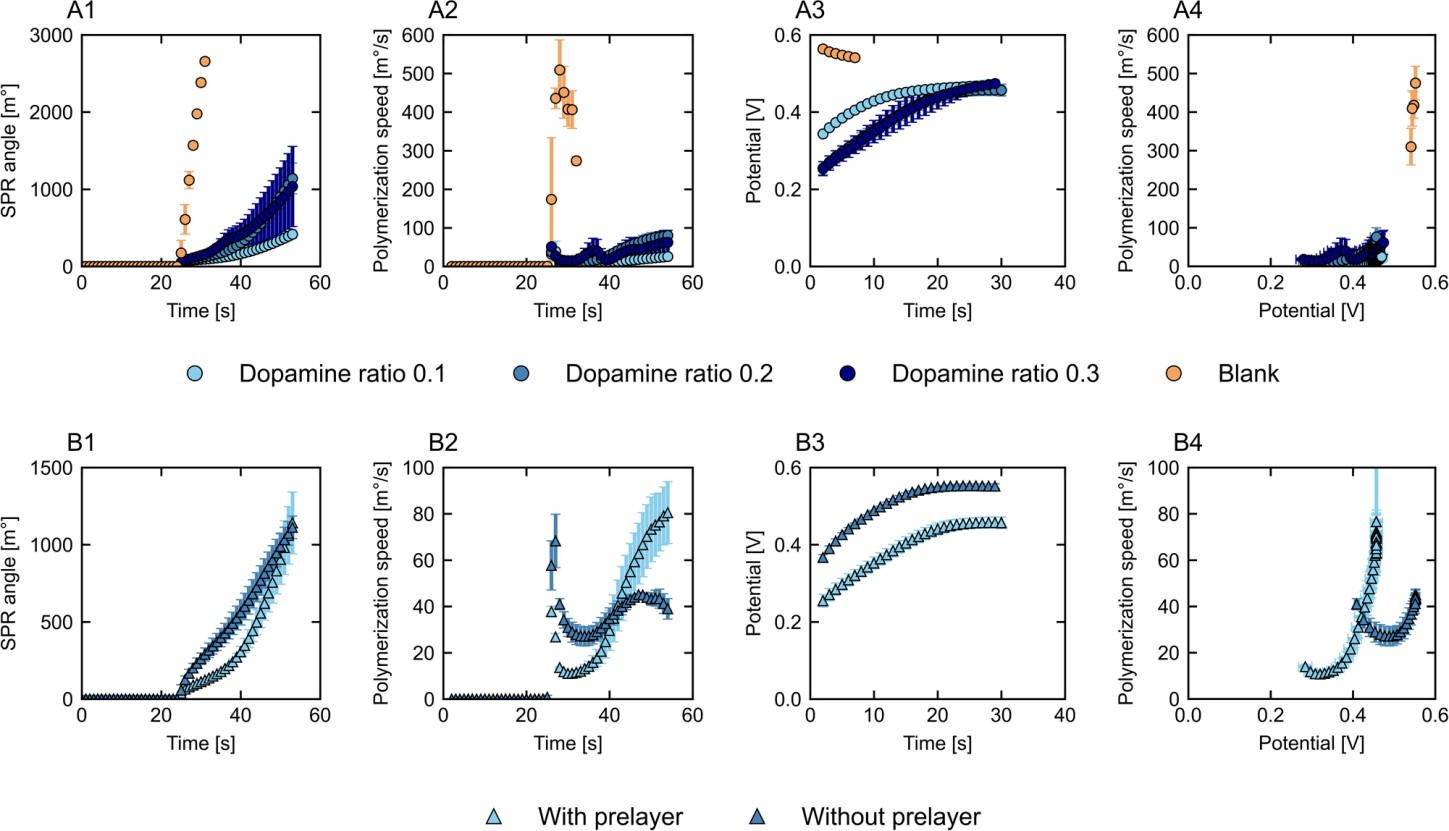
Effect of a dopamine concentration (A) and dopamine prelayer (B) on the SPR curves (1), polymerization speed over time (2), measured potential/current over time (3), and polymerization speed over measured potential/current (4).

To explore the role of dopamine directly at the electrode surface, a homogeneous (poly)­dopamine prelayer was created on the gold electrode surface by exposing it to a dopamine solution at pH 8.5 for 10 min (Figure S6 of the Supporting Information).^57^ Afterward, galvanostatic polypyrrole deposition took place at 50 μA for 30 s (Figure 5B). The prelayer decreases the initial growth of polypyrrole, albeit that after 15 s the polymerization speed increases and the growth becomes faster compared to the surface without the prelayer. This underlines the catalytic role of dopamine during the initiation of polypyrrole polymerization. The slower start is likely related to the insulating effects of the polydopamine prelayer as reflected in the higher measured potential.^57^

In general, there is a clear role for (poly)­dopamine during the initiation of polypyrrole electrodeposition, irrespective of the used deposition technique. The measured current and potential over time indicate a decreased system resistance, which is in line with the catalytic role of dopamine during polypyrrole synthesis. Oxidation products of dopamine, dopaquinone, and dopaminochrome can interact with pyrrole monomers and reduce the conjugation energy of the pyrrole ring, making pyrrole radical formation easier.^37,41^ Although dopamine is thought to have a catalytic role during polypyrrole formation, the measured polymerization amount and speed in the SPR are low in comparison to the blanks. Besides the polymerization reaction itself, dopamine seems to affect the polymerization location. In the presence of dopamine, the formation of colloidal polypyrrole–polydopamine particles in the solution becomes preferential over polypyrrole formation on the gold surface.^41,42^ This reasoning is supported by polymerization experiments on a larger scale, whereby we observed that the synthesis solution becomes black when dopamine is present during electrodeposition.

### Polypyrrole Thickness

In Figure 6, the final SPR angle shift is plotted against the film thickness measured with ellipsometry. The SPR angle shift seems to correlate with the ellipsometry thickness, regardless of the deposition technique and synthesis conditions used, albeit that the spread enlarges at an increasing angle shift. Based on these results, we assume that the measured angle shift during synthesis gives an acceptable indication for the film thickness.

**Figure 6:**
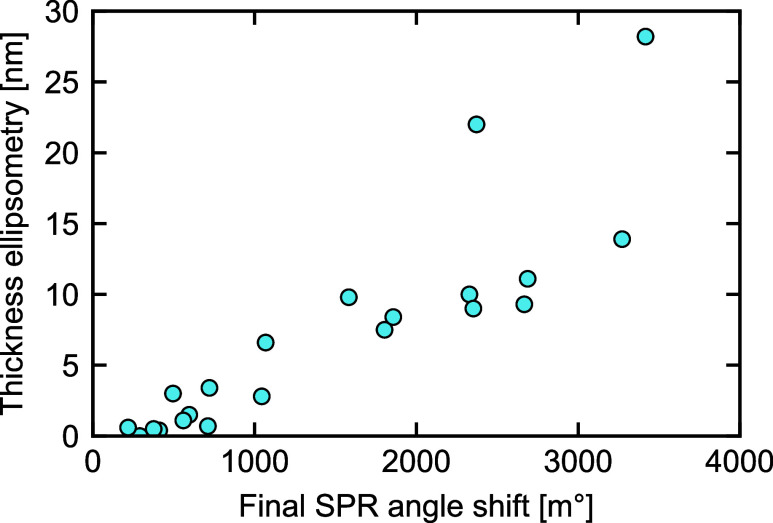
Thickness determined by ellipsometry versus the measured SPR angle shift during polypyrrole synthesis. For all tested synthesis conditions, a sample with and without dopamine is analyzed.

The polypyrrole growth has frequently been linearly correlated to the passed electrical charge.^14,47^ Here, we assessed whether this is valid for our experiments using the SPR angle shift as a measure for thickness. The results, presented in Figure S7 of the Supporting Information, show that there is no overall relation for the techniques investigated here, although within some techniques, this may still be the case. While the charge-based thickness calculation is a convenient approach, we do not advise using it as such without further verification (especially given the differences for samples prepared with and without dopamine).

### Characterization of Polypyrrole

#### Polypyrrole Structure and Heterogeneity

The growth uniformity of polypyrrole over the gold electrode was assessed visually and by SEM analysis. Figure 7 shows images of polypyrrole films prepared with the different deposition techniques, with (A–D) and without (E–H) dopamine present during synthesis. When the samples without dopamine are compared, pulsed deposition techniques result in a more homogeneous appearance. This can be related to smaller polypyrrole particles with a narrower molecular weight being formed during the short on-period.^9,32–34^ The following off-period could enhance reorientation of the already grown polypyrrole,^32^ therewith aiding the more homogeneous appearance. For dopamine-assisted polymerization, static electrodepositions resulted in more homogeneous films on the gold surface. A similar result is shown by Kim et al.,^41^ who reported improved deposition homogeneity for comparable potentiostatic deposition when dopamine was included. The polypyrrole films with dopamine created by pulsed polymerization show more extensive polymerization at the outside compared to that at the middle, with the middle being homogeneous. To the best of our knowledge, this behavior has not been reported previously.

**Figure 7:**
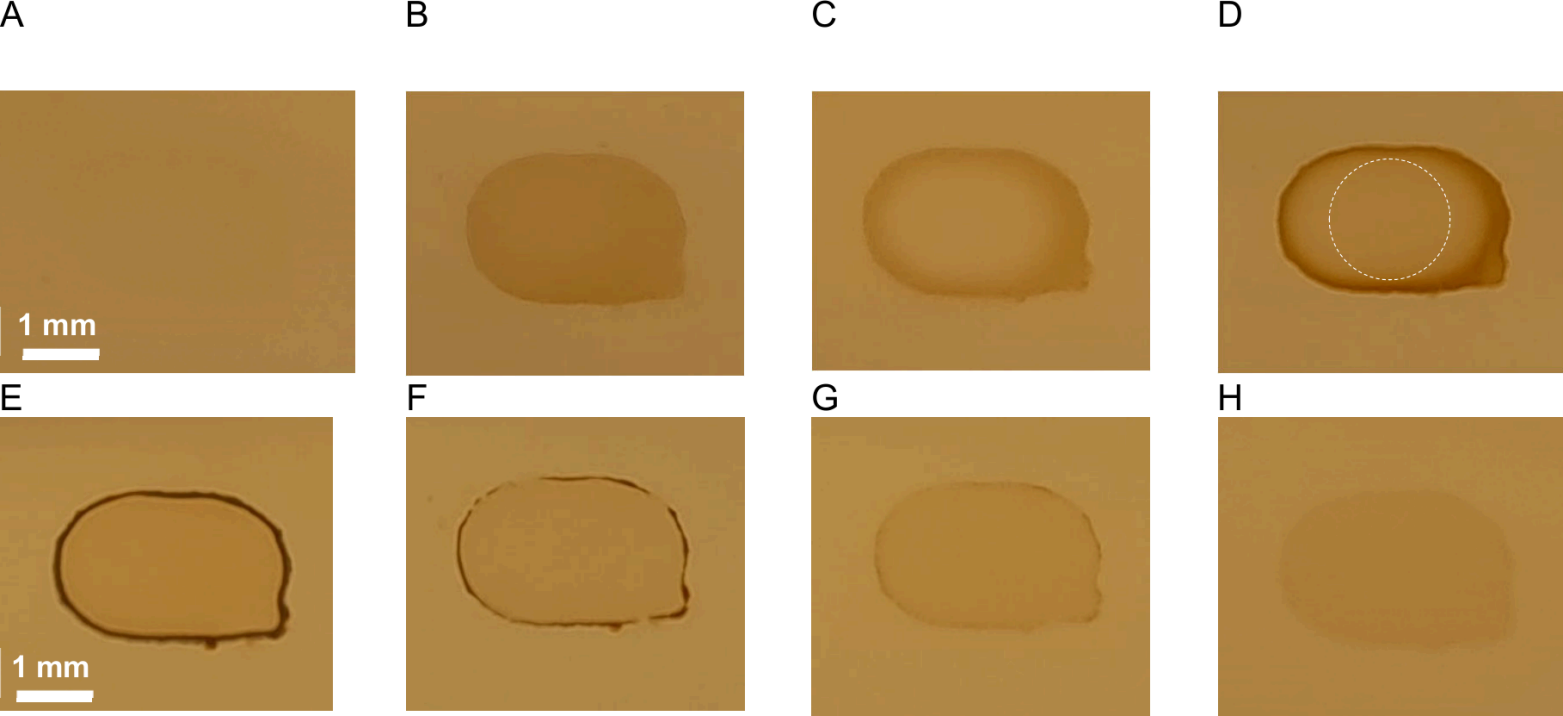
Visual representation of polypyrrole films on gold electrodes with (A–D) and without (E–H) dopamine prepared by galvanostatic (50 μA, A and E), potentiostatic (0.5 V, B and F), pulsed galvanostatic (100 μA, C and G), and pulsed potentiostatic (0.5 V, D and H) deposition. The white circle in D represents the measurement area of the SPR.

It is good to point out that the polymerization time varies greatly for synthesis with or without dopamine, therefore complicating comparison between the final polypyrrole films. Still, there are some effects that are worth mentioning, especially in relation to the SPR results presented earlier. In the visuals in Figure 7, the pulsed techniques seemingly show a thicker, darker polypyrrole film when dopamine is used, while the SPR results indicate less growth. This could be related to the measurement location of the SPR angle in the middle of the gold electrode, illustrated by the white circle in Figure 7D, which does not take the growth at the edges into account. Based on this assessment, we do feel that the SPR data gives a representative impression of the major part of the polymer layers.

SEM was used to visualize the polypyrrole structure formed with the different electrodeposition techniques (Figure 8) for polypyrrole films created at longer deposition times in the presence of dopamine (the other layers were too thin to measure). Because of the relatively long reaction times, homogeneity differences are enlarged (see insets of Figure 8). Potentiostatic deposition, either pulsed or continuous, gives a homogeneous film, even more pronounced than that in Figure 7. Pulsed galvanostatic deposition resulted in a semi-homogeneous center, while the galvanostatic deposition is completely inhomogeneous. This latter observation is surprising as galvanostatic depositions at shorter synthesis times seemed visually very homogeneous (Figure 7) and is most likely related to the extended reaction times.

**Figure 8:**
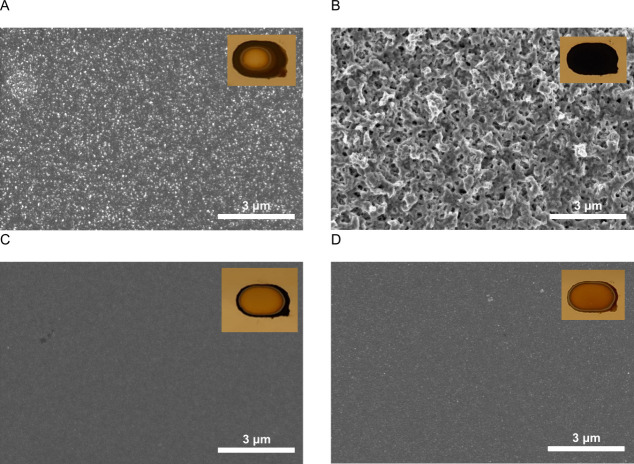
Structure of polypyrrole layers prepared by different deposition techniques: galvanostatic (50 μA, 300 s, A), potentiostatic (0.5 V, 150 s, B), pulsed galvanostatic (150 μA, 1500 cycles, C), and pulsed potentiostatic (0.5 V, 1000 cycles, D). The added pictures provide an idea of the film homogeneity.

Galvanostatic and potentiostatic deposition lead to a uniform globular microstructure (Figure 8), which is coarser for the potentiostatic sample. The difference between these two samples is also described by Wolfart et al.,^28^ and the irregular surface for potentiostatic deposition is described by Du et al.^32^ Polypyrrole films from pulsed techniques reveal a more compact and dense structure (Figure 8C and D). The structure is possibly composed of very small globular structures at a smaller scale compared to the non-pulsed films. Pulsed synthesis leads to polypyrrole particles with a lower molecular weight and narrower molecular weight distribution.^9,33,34^ During the off period, reorientation of attached polypyrrole chains may take place, enhancing conjugation before the next pulse. During the next pulse, nucleation of polypyrrole at new sites becomes more probable.^58^ This more homogeneous nature has also been reported by others.^9,32–34,39^ For static deposition, the polypyrrole can grow by polymerization of monomers with existing chains rather than nucleation at new sites, which results in more irregular growth.^32^

There is a large structural variety in the polypyrrole films, as can be found in the literature, with dependency shown for pH,^27^ electrode surface,^10^ dopamine,^37,41^ and the dopant.^14,18^ Making a clear link between synthesis and structure is rather impossible, although we feel that the initial effects that we report on here are the basis for further understanding of formed structures. Overall, the large diversity of formed structures is a testament to the possibilities of this material.

### Polypyrrole Composition

XPS was used for elemental analysis of the thin polypyrrole films. Figure S8 shows the wide-region XPS spectra for galvanostatic (50 μA), potentiostatic (0.5 V), pulsed galvanostatic (100 μA), and pulsed potentiostatic (0.5 V) deposition. All samples look similar and show the presence of oxygen (O 1s at ∼532 eV), nitrogen (N 1s at ∼400 eV), carbon (C 1s at ∼285 eV), and gold (Au 4d at ∼335 eV). The fact that gold is clearly present is indicative of thin polymer films. When the fraction of gold was excluded, it resulted in an average carbon % of 76.2%, nitrogen % of 4.1%, and oxygen % of 19.1% , with some differences between the deposition conditions. For (pulsed) potentiostatic depositions, the oxygen % increased with the potential (Figure 9A), while for (pulsed) galvanostatic deposition, the oxygen % decreased with the current density (Figure 9B). A higher oxygen % can be caused by a higher level of oxidation^28^ or enhanced inclusion of polydopamine or the dopant. A change in applied potential can affect the attraction of dopant molecules, therewith affecting their incorporation.^13^ Overoxidation is unlikely as the applied or measured potential is below the overoxidation potential,^12^ although oxygen adsorption can take place after polymerization depending on the structure of the film.^13^ The nitrogen % is rather comparable for all samples.

**Figure 9:**
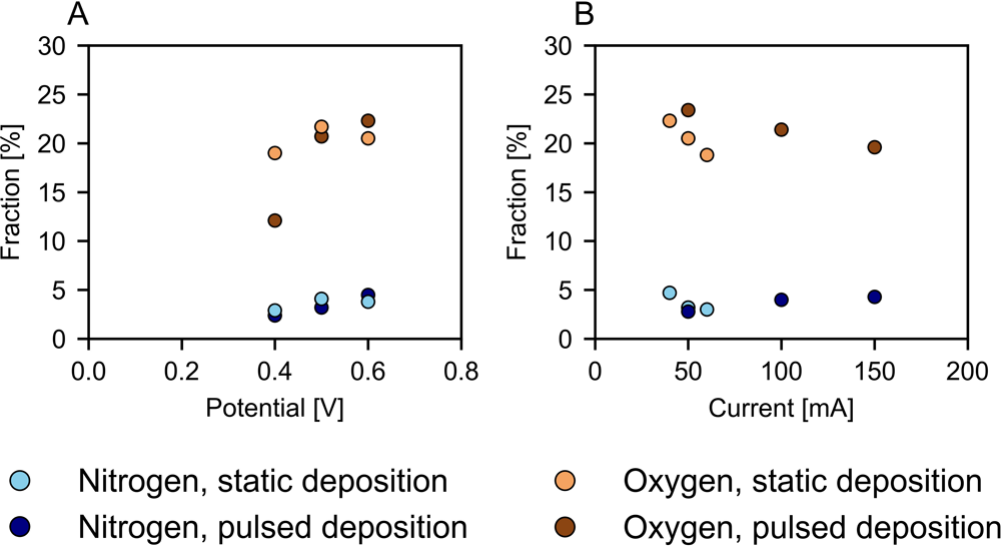
Nitrogen and oxygen content, calculated from XPS analysis, for polypyrrole films prepared through potentiostatic (A), pulsed potentiostatic (A), galvanostatic (B), and pulsed galvanostatic deposition (B).

When the XPS spectra of films without and with dopamine at a 0.2 molar ratio to pyrrole are compared (XPS wide scan can be found in Figure S9), as is done in Figure 10, it can be seen that, for all techniques, the oxygen content increased and nitrogen content decreased when dopamine was present. The corresponding decrease in the N/O ratio can be linked to the incorporation of (poly)­dopamine, which contains 2 oxygen atoms to 1 nitrogen atom per unit, compared to polypyrrole containing no oxygen and 1 nitrogen atom per unit. One could argue that improved incorporation of the dopant could increase the N/O ratio, with 3 oxygen atoms per unit, but this is unlikely as dopamine has been thought to partially replace the dopant.^59^ The drop in the N/O ratio is found for a dopamine ratio of 0.1 and 0.2, while at 0.3, the nitrogen content increased and oxygen content decreased (Figure S10 of the Supporting Information). Keeping all previous aspects in mind, the 0.2 ratio seems most appropriate, which is in accordance with results found by others.^41,59,60^

**Figure 10:**
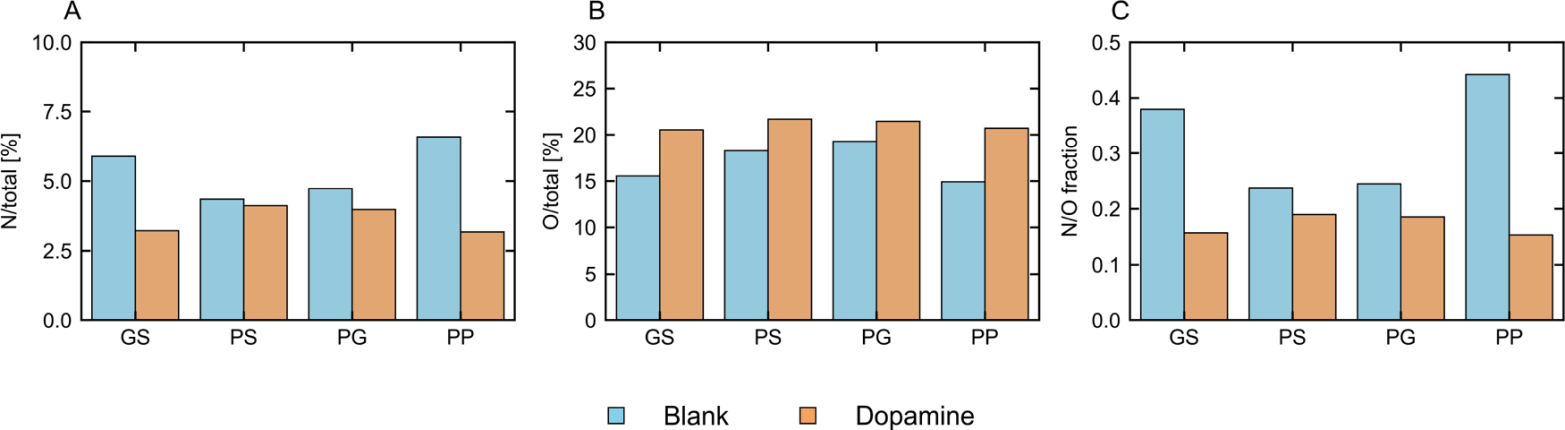
Oxygen % (A), nitrogen % (B), and nitrogen/oxygen ratio (C), calculated from XPS analysis, for polypyrrole films prepared without and with 0.02 M dopamine. GS, galvanostatic; PS, potentiostatic; PG, pulsed galvanostatic; and PP, pulsed potentiostatic.

The narrow spectrum of the C 1s region is shown in Figure 11A. The signals were assigned as follows: ∼285 eV to C–C, ∼286 eV to C–O/C–N, ∼288 eV to C=O/C=N, and ∼289 eV to N–C=O/O–C=N.^61^ The differences in peak height for the deposition techniques and dopamine inclusion indicate that the composition can be altered through the deposition technique, although conclusions on the exact roles that the reaction parameters play cannot be drawn. More in-depth verification experiments and repetitions will be needed to achieve this.

**Figure 11:**
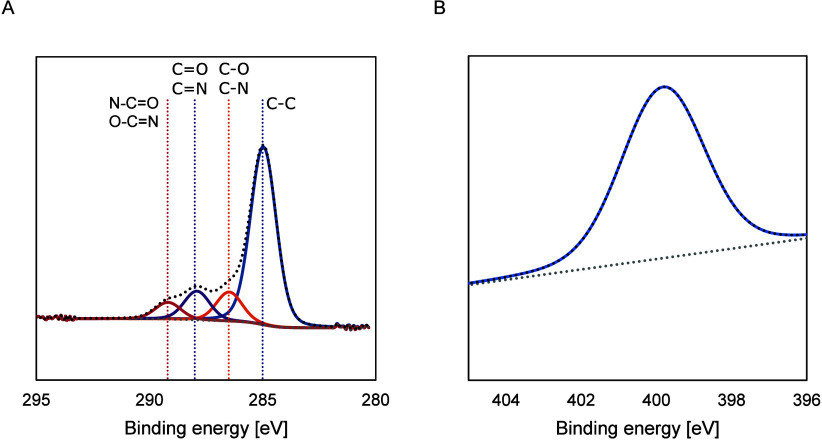
XPS spectra of C 1s (A) and N 1s (B) regions for polypyrrole films prepared with pulsed potentiostatic deposition (0.6 V). Similar spectra were obtained for other deposition techniques.

Figure 11B shows the deconvolution of the N 1s peak. A signal at ∼400 eV is attributed to the −NH group in the pyrrole unit, with C=N defects at ∼398 eV, and the polaron (C–N^+^) and bipolaron (C=N^+^) structures visible at ∼401 and ∼402 eV.^61^ For most of our samples, only one peak was visible around 400 eV, with a slight variation between deposition techniques and dopamine concentrations. Only for the pulsed galvanostatic deposition at 50 μA, a second peak was seen at 398 eV, which in the first instance did not seem logical given the limited growth, yet this may also reflect defects present in that layer.

Both the deposition method and the dopamine concentration influence the final polypyrrole composition, which is mostly reflected in the oxygen content. This is expected to be related to the extent of oxygen reactions after polymerization and/or the incorporation of dopamine during polymerization.

## Conclusions

In this study, we explored the early stages of polypyrrole electrodeposition in real-time with SPR. Polypyrrole films were produced through galvanostatic, potentiostatic, and pulsed deposition, allowing control over the final polymer thickness and polymerization speed, which could be connected to the measured current or potential. Effective control over polypyrrole polymerization could be achieved across all deposition methods when the potential was maintained below 0.6 V. Dopamine was shown to act as a reaction catalyst during the initial polymerization steps, by enhancing pyrrole radical formation at the surface, but the polymerization location moved from surface- to solution-based. This considerably decreased the polymerization speed for all of the depositions.

The deposition method and presence of dopamine influenced the composition, structure, and homogeneity of the polypyrrole films. These insights on initial polypyrrole polymerization and its controllability can be used to design material properties, which is essential for their application as a smart material.

## Supplementary Material



## Notes

Overview of the SPR system, SPR correlation, additional information of pulsed depositions, potential–time and polymerization speed–potential graphs for dopamine copolymerization, formation of the (poly)­dopamine prelayer, polypyrrole growth versus passed charge, XPS wide spectra for different deposition techniques, XPS wide spectra for varying dopamine/pyrrole ratios, and oxygen % and nitrogen % for dopamine-containing polypyrrole films (DOCX)
