# Supplementary material for: Real-Time Polypyrrole Electrodeposition Kinetics Monitored by Surface Plasmon Resonance: Impact of the Deposition Method and Dopamine Copolymerization
Source: Langmuir. 2025 Jul 18;41(29):19332–43. doi: 10.1021/acs.langmuir.5c01765 (PMC12312161; doi:10.1021/acs.langmuir.5c01765)
Supplement: Supplementary file 1 [file la5c01765_si_001.docx]

**Supporting Information**

Real-time polypyrrole electrodeposition kinetics monitored by surface plasmon resonance: Impact of deposition method and dopamine co-polymerization

Kieke de Boer*, Karin Schroën*

Laboratory of Food Process Engineering, Wageningen University & Research, Bornse Weilanden 9, 6708 WG Wageningen, the Netherlands^[[1]](#footnote-1)^

Number of pages: 10
Number of figures: 10
Number of schemes: 0
Number of tables: 0

**Contents**

**Figure S1.** Photo of the used SPR system (A) and schematic of the electrochemical cell with insert of a gold sensor electrode (WE) (B).

**Figure S2.** The correlation between the SPR angle shift between point 1-5 and point 2-4 from Figure 1.

**Figure S3.** Example potential-time, current-time, and SPR angle shift-time curves for pulsed galvanostatic (A, C, E) and pulsed potentiostatic deposition (B, D, F), respectively.

**Figure S4.** Measured potential (A/C) and current (B/D) for polypyrrole deposition in the presence and absence of 0.02 M dopamine. Galvanostatic (A), potentiostatic (B), pulsed galvanostatic (C), and pulsed potentiostatic (D) deposition.

**Figure S5.** Polymerization speed versus the measured potential (A/C) or current (B/D) for polypyrrole deposition in the presence and absence of 0.02 M dopamine. Galvanostatic (A), potentiostatic (B), pulsed galvanostatic (C), and pulsed potentiostatic (D) deposition.

**Figure S6.** Average SPR curve of dopamine self-polymerization and deposition on the clean SPR gold sensor surface for 10 minutes. The average result of 4 replicates is shown here, which standard deviation in light blue.

**Figure S7.** Final polypyrrole growth for different deposition techniques plotted against the passed charge (Q) during synthesis. Galvanostatic (A), potentiostatic (B), pulsed galvanostatic (C), and pulsed potentiostatic (D) deposition.

**Figure S8.** Wide region XPS spectra for galvanostatic 50µA (GS), potentiostatic 0.5 V (PS), pulsed galvanostatic 100 µA (PG), and pulsed potentiostatic 0.5 V (PP) deposition.

**Figure S9.** Wide region XPS spectra for galvanostatic polypyrrole deposition in the absence of dopamine or in the presence of dopamine in a ratio of 0.1, 0.2, or 0.3 compared to pyrrole (blank).

**Figure S10.** The oxygen% (⬤), nitrogen% (⬤), and nitrogen/oxygen ratio % (⬤), calculated from XPS analysis, for polypyrrole films prepared at varying dopamine : pyrrole ratios.

| A | B |
| --- | --- |
| *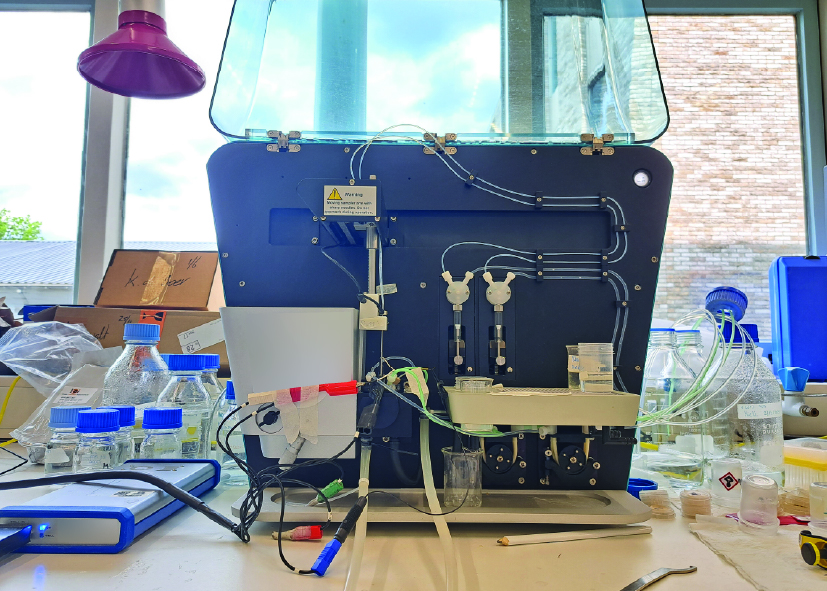* | ***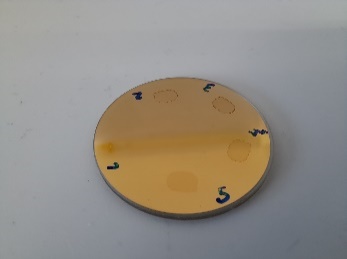***  ***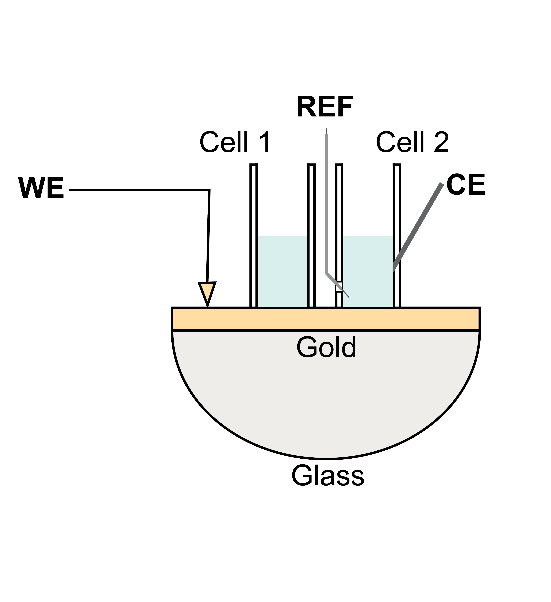*** |

**Figure S1.** Photo of the used SPR system (A) and schematic of the electrochemical cell with insert of a gold sensor electrode (WE) (B).


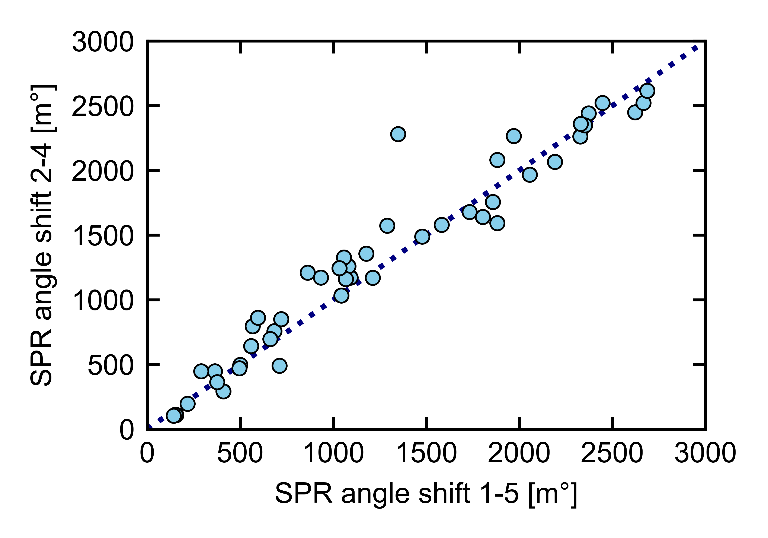


**Figure S2.** The correlation between the SPR angle shift between point 1-5 and point 2-4 from Figure 1.

| A | B |
| --- | --- |
|  |  |
| C | D |
|  |  |
| E | F |
| 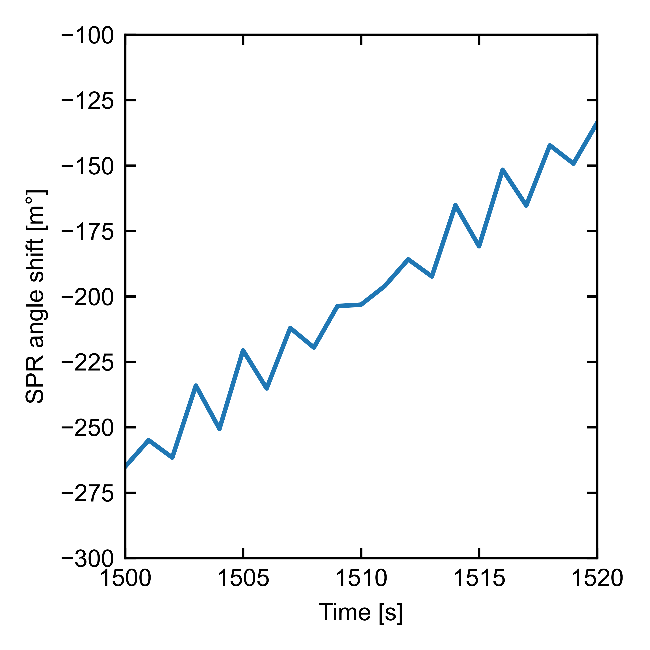 | 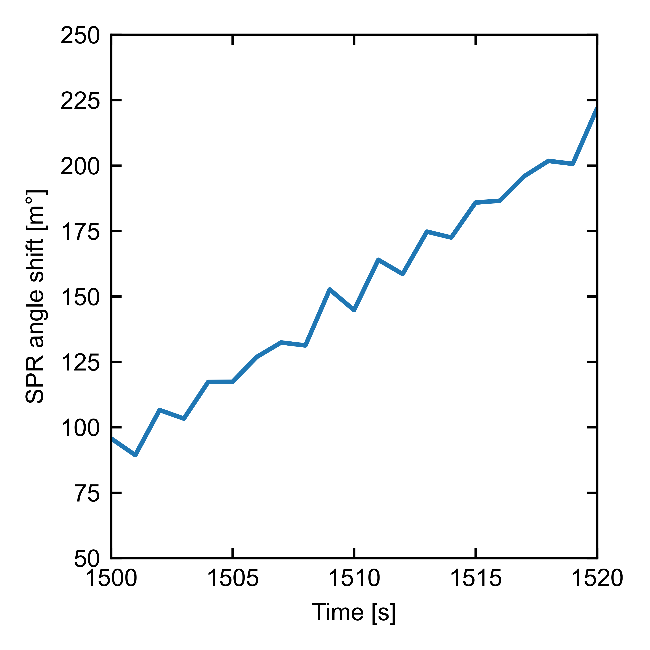 |

**Figure S3.** Example potential-time, current-time, and SPR angle shift-time curves for pulsed galvanostatic (A, C, E) and pulsed potentiostatic deposition (B, D, F), respectively.


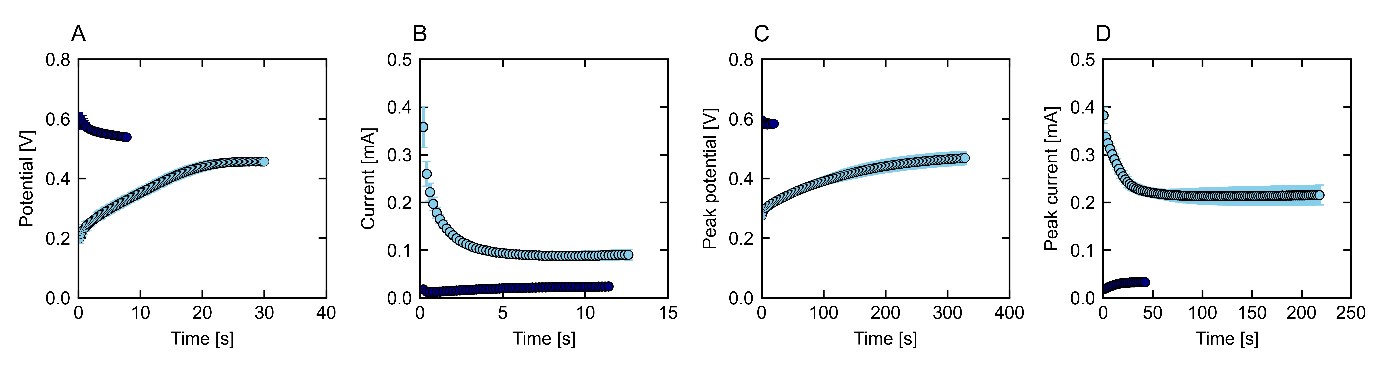


*
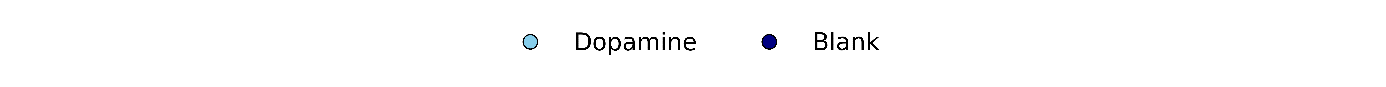
*

**Figure S4.** Measured potential (A/C) and current (B/D) for polypyrrole deposition in the presence and absence of 0.02 M dopamine. Galvanostatic (A), potentiostatic (B), pulsed galvanostatic (C), and pulsed potentiostatic (D) deposition.


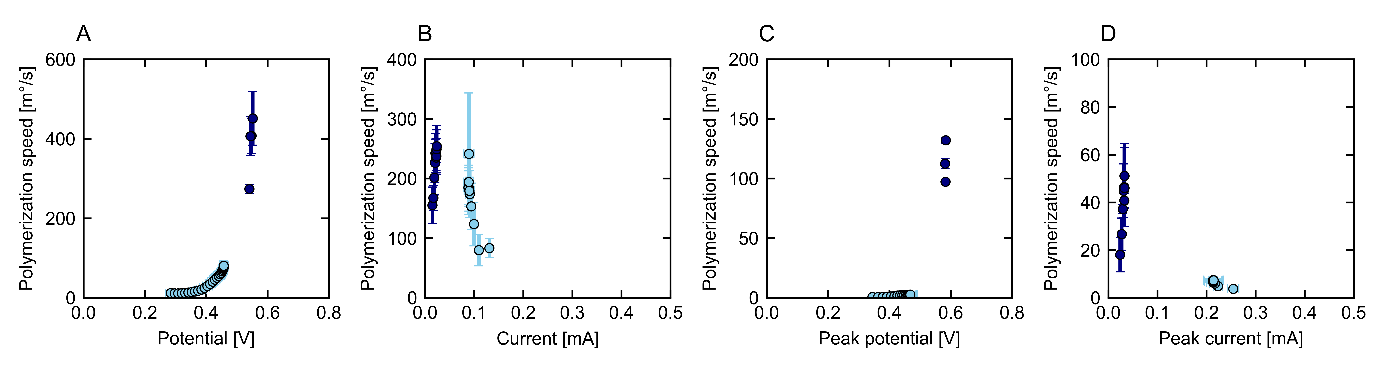


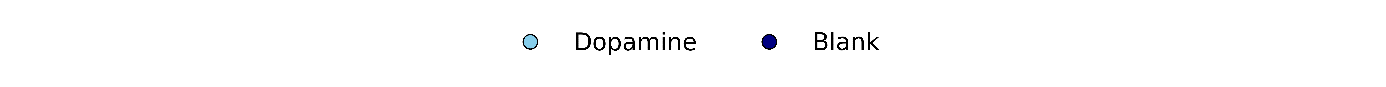


**Figure S5.** Polymerization speed versus the measured potential (A/C) or current (B/D) for polypyrrole deposition in the presence and absence of 0.02 M dopamine. Galvanostatic (A), potentiostatic (B), pulsed galvanostatic (C), and pulsed potentiostatic (D) deposition.

**Figure S6.** Average SPR curve of dopamine self-polymerization and deposition on the clean SPR gold sensor surface for 10 minutes. The average result of 4 replicates is shown here, with standard deviation in light blue.


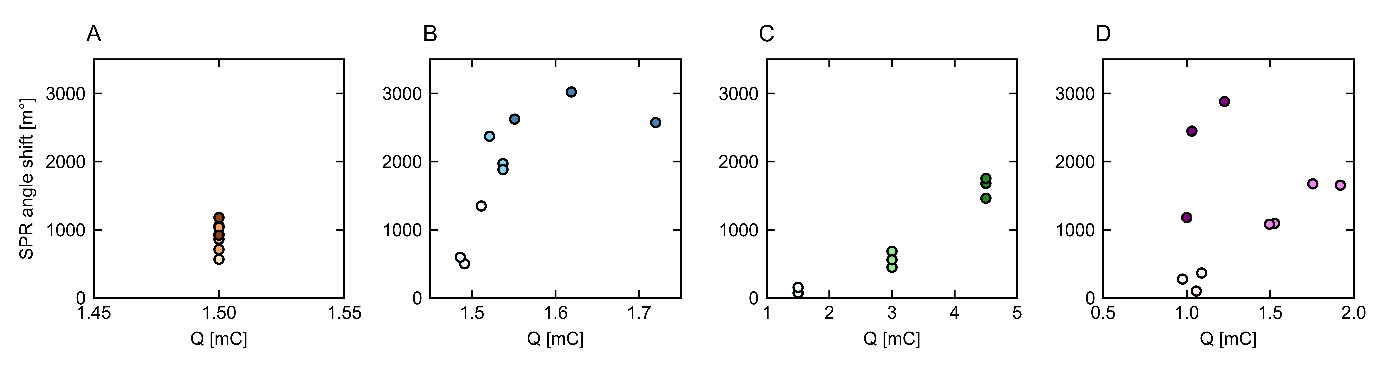


| ⬤ 40 μA ⬤ 50 μA ⬤ 60 μA | ⬤ 0.4 V ⬤ 0.5 V ⬤ 0.6 V | ⬤ 50 μA ⬤ 100 μA ⬤ 150 μA | ⬤ 0.4 V ⬤ 0.5 V ⬤ 0.6 V |
| --- | --- | --- | --- |

**Figure S7.** Final polypyrrole growth for different deposition techniques plotted against the passed charge (Q) during synthesis. Galvanostatic (A), potentiostatic (B), pulsed galvanostatic (C), and pulsed potentiostatic (D) deposition.


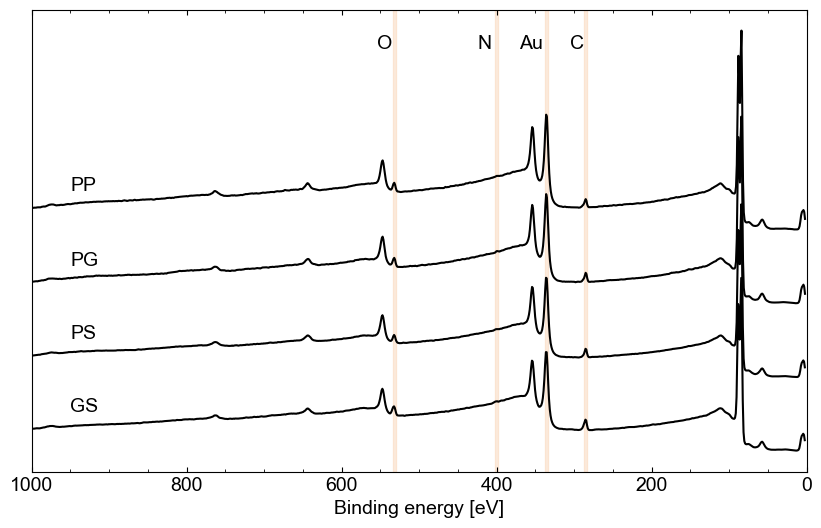


**Figure S8.** Wide region XPS spectra for galvanostatic 50µA (GS), potentiostatic 0.5 V (PS), pulsed galvanostatic 100 µA (PG), and pulsed potentiostatic 0.5 V (PP) deposition.


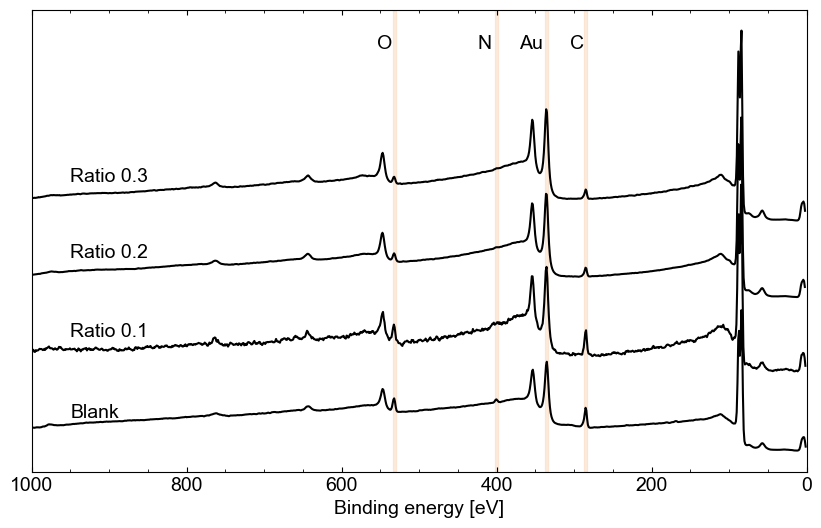


**Figure S9.** Wide region XPS spectra for galvanostatic polypyrrole deposition in the absence of dopamine or in the presence of dopamine in a ratio of 0.1, 0.2, or 0.3 compared to pyrrole (blank).

**Figure S10**. The oxygen% (⬤), nitrogen% (⬤), and nitrogen/oxygen ratio % (⬤), calculated from XPS analysis, for polypyrrole films prepared at varying dopamine : pyrrole ratios.

1. *Email: [Karin.schroen@wur.nl](mailto:Karin.schroen@wur.nl), Kieke.deboer@wur.nl [↑](#footnote-ref-1)
